# The influence of anti-smoking messages to Indonesian youth smoking behavior: the Indonesian 2019 Global Youth Tobacco Survey (GYTS)

**DOI:** 10.1186/s12889-023-15830-5

**Published:** 2023-05-19

**Authors:** Hario Megatsari, Rita Damayanti, Dian Kusuma, Tati Suryati Warouw, Siti Rahayu Nadhiroh, Erni Astutik, Desak Made Sintha Kurnia Dewi, Susy Katikana Sebayang

**Affiliations:** 1 Faculty of Public Health, Universitas Airlangga, Surabaya, Indonesia; 2 Faculty of Public Health, Universitas Indonesia, Jakarta, Indonesia; 3 Department of Health Services Research and Management, School of Health & Psychological Sciences, University of London, London, UK; 4 Indonesia National Research and Innovation Agency, Jakarta, Indonesia; 5 School of Health and Life Sciences, Universitas Airlangga, Banyuwangi, Indonesia

**Keywords:** Exposure, Anti-smoking messages, Youth, Tobacco control, Public health

## Abstract

**Introduction:**

Various anti-tobacco promotions have emerged in order to reduce the detrimental impacts of tobacco advertising on adolescents. The objective of this study is to explore the relationship between the exposure to anti-smoking messages and Indonesian youth smoking behavior.

**Method:**

We used secondary data from the Indonesian 2019 Global Youth Tobacco Survey (GYTS). The participants were students from grades seven to twelve. We used multiple logistic regression to assess the relationship of anti-smoking messages exposure on the smoking behavior variable. We used complex samples process logistic regression to calculate odds ratios (ORs) and 95% confidence intervals (CIs) and controlling for relevant covariables.

**Results:**

The percentage of the exposure to anti-smoking messages in all types were not more than 25% in each outcome variables. The results also showed that in the current smoker variables, adolescent who exposed to the two variables of anti-smoking messages increased the odds to become current smoker. The variables were anti smoking messages in media (AOR 1.41; 95% CI 1.15–1.73) and in school (AOR 1.26; 95% CI 1.06–1.50). On the other hand, in the smoking susceptibility variables, there were no variables of anti-smoking messages that had relation with it.

**Conclusions:**

The study concluded that there were only two variables of the anti-smoking messages that had relation with the Indonesian youth smoking behavior, which were current smokers. Unfortunately those variables increased the odds of the respondents to become current smokers. Indonesia government should develop media following international best practices to convey the anti-smoking messages.

## Introduction

By 2019, approximately 1 billion people worldwide used tobacco products, including 847 million men and 153 million women [1]. A total of 25 million of these tobacco users are teenagers aged 13 to 15 [2]. The Southeast Asia Region (SEARO) and Western Pacific Region (WPRO) have the most smokers, accounting for approximately 6.4 million and 4.7 million, respectively [2]. Indonesia is a major contributor to the region’s number of smokers [2].

In Indonesia, adolescents aged 13 to 18 had a smoking prevalence of about 38.3% [2]. This percentage was significantly higher than in neighboring Malaysia (20.6%), Thailand (17.2%), and Myanmar (17%) [2]. Indonesia also had the youngest of age smoking initiation among the Association of Southeast Asia Nation (ASEAN) countries, which was around 16.8 years old [2, 3]. As a result, the Indonesian government must implement best practices in tobacco control in order to reduce the burden of tobacco-related diseases.

A large number of studies indicates that smoking at a young age has a negative impact on health instantly and increases the development of chronic diseases throughout one’s life [4]. Cigarette smoking in youth can lead to nicotine addiction, which has a long-term negative impact on brain development [5]. Furthermore, young smokers are at risk of slowing lung function and impaired lung growth [5]. An earlier case–control research in Blitar City found that smoking before the age of 15 increased the risk of getting Chronic Obstructive Pulmonary Disease (COPD) by 12 times compared to not smoking at all [6].

In addition, tobacco industries use both direct and indirect marketing efforts, such as sponsorship of sporting events and music festivals (e.g. billboards and commercials) [4]. Previous research have conclusively shown a relationship between teenage smoking behavior and tobacco advertisement [7–9]. Various anti-tobacco advertising have appeared in an effort to lessen the negative effects tobacco promotions have on youth. Centers for Disease Control and Prevention (CDC) stated that there were four health communication strtategies to convey anti-smoking messages, which were paid media, earned media, social media, and program communication [10]. Most advertising have emphasized negative health implications, defying social forces and influences, or the tobacco industry’s financial interests and other aspects [11–13].

Several researches looked at how anti-tobacco media affected teenage tobacco use reduction or prevention [14, 15]. Other researches related to anti-tobacco media focused on the anti-smoking initiatives and current smoking status in order to design effective interventions to curtail the smoking epidemic [16]. In 2014, a study conducted by Minh et al., analyzed the access to antismoking information among school children aged 13 to 15 years in Vietnam and examined its potential impact on preventing smoking initiation [17]. Several researches found that anti-smoking messages can be conveyed through various means. For example, study conducted by Huang et al., (2018) found that testimonial commercials with visual and emotionally evocative depictions of smoking-related ailments, may have a stronger effect on encouraging Taiwanese smokers to quit smoking [18]. Another example was a study conducted by Manocci et al., [19] showed that teenagers prefer anti smoking messages with scientific profile and those who uncover incorrect preconceptions about smoking.

However, few utilized secondary data to investigate youth smoking behavior and anti-smoking media in the national context. The secondary data’ analysis will be beneficial for the government to make adjustment in the public health policy and public health programs. Therefore, our objective was to to explore the relationship between the exposure to anti-smoking messages and adolescents’ smoking behavior based on the Indonesian 2019 Global Youth Tobacco Survey (GYTS).

## Methods

### Data source

The Indonesian GYTS 2019 was a cross-sectional study undertaken in Indonesia’s public and private schools to evaluate tobacco use among students aged 13 to 17. The authors obtained the Indonesia GYTS 2019 data from the CDC website: https://nccd.cdc.gov/GTSSDataSurveyResources/Ancillary/DataReports.aspx?CAID=2.

The sample for the survey was divided into two phases: the first phase was choosing schools with probability proportionate to size (PPS). The second stage was selecting classes at random from different schools. The whole student population from the chosen classes was surveyed [20].

Sample size calculations were carried out using standard methods by the CDC, Atlanta. Sample distribution divided into 3 regions namely Java, Sumatra and others. Each region is selected 25 junior high schools and 25 high school schools per region, and each school was randomly selected as a class with a random number assigned to each school. Total sampling 150 schools, located in 30 provinces.

The sample size from each school will differ according to the number of classes and students in each school. All students in the class selected as the sample will took part in the survey and distributed questionnaires and answer sheets. The predicted number of samples matched inclusion criteria of around 10,500 students. All students in the sampled class will as respondents (total sample will be more than 10,500 students).

The process of data collection begun by the enumerators had a small discussion with the teacher team and administration at school, explained the sample class, and all students in the class took part in the survey. The interview will be conducted on all students who in the selected class, with an estimated time of 45 min. All equipment surveys (questionnaires, answer sheets, stationery and erasers) were provided by the research team.

Before the questionnaire is distributed to each student, the enumerators will explain about the procedures for filling out answer sheets and the questionnaire. Each student will be asked for approval to participate in surveys. The data collected is the primary data resulting from the answer choices immediately from every student. After completing all the questionnaires the answer sheets will be collected and brought by the research team.

### Variables

We used three variables in this study: outcome variables, covariates, and independent variables. The first variable was outcome variables that consisted current smoking behavior and smoking susceptibility. The next variable was covariates that consisted of characteristic of the respondents, accessibility to buy cigarettes, second hand smoking, and tobacco advertisement, promotion and sponsorship (TAPS) exposure. And the last variable was independent variables that consisted of anti-smoking messages in media, at events, and at schools. The description of each variables were described in the Table 1.

**Table 1:** Variables of the study

No	Variables	Original questions Indonesian GYTS 2019	Measurements
**1**	**Outcomes**		
**1.a**	**Current Smoker**	During the past 30 days, on how many days did you smoke cigarettes?	Respondents who responded with a value of **0** were classified as non-smokers, while those who responded with a value of **1 or more** were classified as current smokers
**1.b**	**Smoking susceptibility**	1. If one of your best friends offered you a cigarette product, would you use it? 2. At any time during the next 12 months do you think you will consume cigarette in any form?	Respondents who responded with **“definitely not”** on both questions were classified as non susceptible, while others classified as susceptible
**2**	**Covariates**		
**2.a**	**Characteristics of Respondents**		
2.a.i	Sex	What is your sex?	Respondents answered **Male** or **Female** for this question
2.a.ii	Grade	In what grade/form are you?	Respondents answered number 7, 8, 9, 10, 11 or 12 for this question, and after that we classified number 7, 8, and 9 as **Junior High School** and number 10, 11, and 12 as **Senior High School**
2.a.iii	Weekly spending money	During an average week, how much money do you have that you can spend on yourself, however you want?	Respondents answered: **more than IDR 50,000**, **IDR 41,000–50,000**, **IDR 31,000–40,000**, **IDR 21,000–30,000**, **IDR 11,000–20,000**, **less than IDR 11,000**, or **usually don't have any spending money**, for this question
**2.b**	**Accessibility to buy or consume cigarette**	Can you purchase cigarettes near your school?	Respondents answered **Easy** or **Difficult** for this question
**2.c**	**Second hand smoking**		
2.c.i	Saw anyone smoking in the house	During the past 7 days, on how many days has anyone smoked inside your home, in your presence?	Respondents who responded with a value of **0 day** were categorized as **No**, while those who responded with a value of **1 day or more** were categorized as **Yes**
2.c.ii	Saw anyone smoking in the school	During the past 30 days, did you see anyone smoke inside the school building or outside on school property?	Respondents answered **Yes** or **No** for this question
2.c.iii	Parents smoke tobacco	Do your parents smoke tobacco?	Respondents answered **Yes** or **No** for this question
**2.d**	**Tobacco advertisement, promotion and sponsorship (TAPS) exposure**		
2.d.i	Tobacco advertisement exposure	1. During the past 30 days, did you see any people using tobacco on TV, in videos, or in movies? 2. Over the past 30 days, have you seen cigarette advertisements/promotions/brand names/cigarette logos on television? 3. During the past 30 days, did you see any advertisements for cigarette products in newspapers or magazines? 4. During the past 30 days, did you see any cigarette advertisements in outdoor media? (such as billboards, banners, posters, digital billboards) 5. During the past 30 days, did you see any cigarette advertisements when you opened in the internet or social media? 6. During the past 30 days, did you see any advertisements or promotions for cigarette products in sales centers (such as shops, stalls, kiosks and minimarket)?	Respondents answered Yes or No in each questions, and after that we calculate Yes and No question and classified as **No Exposure**, **One Type Exposure** and **More Than One Exposure**
2.d.ii	Tobacco promotion exposure	1. Would you ever use or wear something that has a cigarette company or cigarette product name or picture on it such as a match, t-shirt, hat, or sunglasses? 2. Do you have something (for example, t-shirt, pen, backpack, hat or sun glasses) with a cigarette product brand logo on it? 3. Has a person working for a tobacco company ever offered you a free tobacco product? 4. Have you ever got free cigarettes/discounted cigarette coupons/vouchers from cigarette companies?	Respondents answered Yes or No in each questions, and after that we calculate Yes and No question and classified as **No Exposure**, **One Type Exposure** and **More Than One Exposure**
2.d.iii	Tobacco sponsorship exposure	1. During the past 30 days, did you see any advertisements for cigarette products when you attended sports events? 2. During the past 30 days, did you see any advertisements for cigarette products at music concerts? 3. During the past 30 days, did you see any advertisements for cigarette products at community events/social gatherings?	Respondents answered Yes or No in each questions, and after that we calculate Yes and No question and classified as **No Exposure**, **One Type Exposure** and **More Than One Exposure**
**3**	**Independents**		
**3.a**	**Anti-Smoking Exposure**		
3.a.i	Anti-smoking messages on media (i.e.: television, radio, internet, billboards, posters, newspapers, magazines, or movies)	During the past 30 days, did you see or hear any anti-cigarette media messages on television, radio, internet, billboards, posters, newspapers, magazines, or movies?	Respondents answered **Yes** or **No** for this question
3.a.ii	Anti-smoking message at events (i.e.: sports events, fairs, concerts, or community events, or social gatherings)	During the past 30 days, did you see or hear any anti-cigarette messages at sports events, fairs, concerts, or community events, or social gatherings?	Respondents answered **Yes** or **No** for this question
3.a.iii	Anti-smoking messages in the school	During the past 12 months, were you taught in any of your classes about the dangers of tobacco use?	Respondents answered **Yes** or **No** for this question

### Statistical analysis

We used complete cases in our study and took the complex sampling plan into account. We assessed the demographic information of the respondents and used multivariable logistic regression to assess the correlation between the results and the independent variables. STATA 16.0 was used to analyze the data.

### Ethical clearance

The Indonesian 2019 GYTS has passed ethical clearance from the Health Research Ethics Commission, National Health Research and Development Agency, with notification number: LB.02.01/2/KE.315/2019. The authors delete all respondents’ identities from the dataset. Respondents have provided written approval for their involvement in the study through filling the informed concent. All methods were performed in accordance with the relevant guidelines and regulations.

## Results

Tables 2 and 3 show that the tolerance value for all variables is greater than 0.10, whereas the VIF value for all variables is smaller than 10.00. According to the multicollinearity test, the test shows that there was no strong association between independent variables in the regression model.

**Table 2:** Results for the co-linearity (Dependent variable: current smoker variable)

**Variables**	**Collinearity statistics**	
	**Tolerance**	**VIF**
Anti-smoking messages on media	0.890343	1.12
Anti-smoking message at events	0.895283	1.12
Anti-smoking messages in the school	0.951813	1.05
Grade	0.946238	1.06
Weekly spending money	0.939882	1.06
Accessibility to buy or consume cigarette	0.954073	1.05
Saw anyone smoking in the house	0.876962	1.14
Saw anyone smoking in the school	0.947435	1.06
Parents smoke tobacco	0.903266	1.11
Tobacco advertisement exposure	0.881795	1.13
Tobacco promotion exposure	0.879809	1.14
Tobacco sponsorship exposure	0.841122	1.19

**Table 3:** Results for the co-linearity (Dependent variable: smoking susceptibility variable)

**Variables**	**Collinearity statistics**	
	**Tolerance**	**VIF**
Anti-smoking messages on media	0.882641	1.13
Anti-smoking message at events	0.896803	1.12
Anti-smoking messages in the school	0.947314	1.06
Grade	0.943694	1.06
Weekly spending money	0.937712	1.07
Accessibility to buy or consume cigarette	0.954606	1.05
Saw anyone smoking in the house	0.880165	1.14
Saw anyone smoking in the school	0.951624	1.05
Parents smoke tobacco	0.889714	1.12
Tobacco advertisement exposure	0.873255	1.15
Tobacco promotion exposure	0.920665	1.09
Tobacco sponsorship exposure	0.872075	1.15

Table 4 showed the distribution of respondents by current smoker and smoking susceptibility. Based on the sex, the percentage of male much greater than female in the both current smoker and smoking susceptibility variables. According to the education level, both of the outcome variables have similar percentage of the result. And for the weekly spending money, the current smoker variables and smoking susceptibility variables had the highest percentage in the group of less than IDR 11,000.

**Table 4:** Distribution of respondents by current smoker and smoking susceptibility

**Variables**	**Current Smoker (** ***n*** ** = 8,428)**					**Smoking Susceptibility (** ***n*** ** = 6,952)**				
	**Non Smoker**		**Current Smoker**		***P*** ** value**	**Non Susceptible**		**Susceptible**		***P*** ** value**
	**%**	**CI**	**%**	**CI**		**%**	**CI**	**%**	**CI**	
**Anti-Smoking Exposure**										
Anti-smoking messages on media (i.e.: television, radio, internet, billboards, posters, newspapers, magazines, or movies)										
Yes	78.6	[75.7,81.2]	21.4	[18.8,24.3]	0.015	86.9	[84.7,88.9]	13.1	[11.1,15.3]	0.298
No	81.8	[79.8,83.7]	18.2	[16.3,20.2]		88.2	[86.9,89.3]	11.8	[10.7,13.1]	
Anti-smoking message at events (i.e.: sports events, fairs, concerts, or community events, or social gatherings)										
Yes	74.1	[70.7,77.2]	25.9	[22.8,29.3]	0.000	86.9	[84.9,88.6]	13.1	[11.4,15.1]	0.160
No	84.1	[82.4,85.6]	15.9	[14.4,17.6]		88.3	[87.1,89.4]	11.7	[10.6,12.9]	
Anti-smoking messages in the school										
Yes	81.3	[79.2,83.2]	18.7	[16.8,20.8]	0.827	88.2	[86.9,89.3]	11.8	[10.7,13.1]	0.495
No	81	[78.4,83.3]	19	[16.7,21.6]		87.5	[85.6,89.2]	12.5	[10.8,14.4]	
**Sex**										
Female	97.7	[96.8,98.4]	2.3	[1.6,3.2]	0.000	92.3	[91.1,93.4]	7.7	[6.6,8.9]	0.000
Male	61.5	[57.6,65.2]	38.5	[34.8,42.4]		79.6	[77.1,81.9]	20.4	[18.1,22.9]	
**Grade**										
Junior High School	81.3	[78.7,83.6]	18.7	[16.4,21.3]	0.842	88	[86.3,89.4]	12	[10.6,13.7]	0.871
Senior High School	80.9	[78.1,83.4]	19.1	[16.6,21.9]		87.8	[86.3,89.1]	12.2	[10.9,13.7]	
**Weekly spending money**										
More than Rp50,000	82.9	[80.3,85.2]	17.1	[14.8,19.7]	0.002	87.7	[85.8,89.4]	12.3	[10.6,14.2]	0.874
Rp41,000-Rp50,000	83.2	[79.7,86.2]	16.8	[13.8,20.3]		87.7	[84.7,90.1]	12.3	[9.9,15.3]	
Rp31,000-Rp40,000	84.8	[80.6,88.2]	15.2	[11.8,19.4]		89.6	[86.4,92.2]	10.4	[7.8,13.6]	
Rp21,000-Rp30,000	83	[79.7,85.9]	17	[14.1,20.3]		87.7	[84.4,90.3]	12.3	[9.7,15.6]	
Rp11,000-Rp20,000	78.8	[76.1,81.3]	21.2	[18.7,23.9]		88.4	[86.4,90.2]	11.6	[9.8,13.6]	
Less than Rp11,000	78.2	[75.5,80.7]	21.8	[19.3,24.5]		87.1	[84.0,89.7]	12.9	[10.3,16.0]	
Usually don’t have any spending money	79.1	[73.1,84.0]	20.9	[16.0,26.9]		88.2	[84.3,91.2]	11.8	[8.8,15.7]	
**Accessibility to buy or consume cigarette**										
Easy	80.4	[78.2,82.4]	19.6	[17.6,21.8]	0.295	89	[87.7,90.1]	11	[9.9,12.3]	0.008
Difficult	81.8	[79.4,84.0]	18.2	[16.0,20.6]		87	[85.5,88.3]	13	[11.7,14.5]	
**Second hand smoking**										
Saw anyone smoking in the house										
Yes	72.6	[70.4,74.6]	27.4	[25.4,29.6]	0.000	85.8	[84.3,87.2]	14.2	[12.8,15.7]	0.000
No	91.6	[90.0,93.0]	8.4	[7.0,10.0]		90.2	[88.7,91.6]	9.8	[8.4,11.3]	
Saw anyone smoking in the school										
Yes	85.4	[83.2,87.3]	14.6	[12.7,16.8]	0.000	90.2	[88.7,91.6]	9.8	[8.4,11.3]	0.000
No	77.6	[75.6,79.5]	22.4	[20.5,24.4]		85.8	[84.3,87.2]	14.2	[12.8,15.7]	
Parents smoke tobacco										
Yes	82.5	[80.4,84.4]	17.5	[15.6,19.6]	0.036	89.3	[87.8,90.6]	10.7	[9.4,12.2]	0.002
No	79.5	[76.9,81.9]	20.5	[18.1,23.1]		86.2	[84.7,87.5]	13.8	[12.5,15.3]	
**Tobacco Advertisement, Promotion and Sponsorship (TAPS) Exposure**										
Tobacco advertisement exposure										
No exposure	89.8	[87.1,91.9]	10.2	[8.1,12.9]	0.000﻿	93.1	[90.6,95.0]	6.9	[5.0,9.4]	0.001
One type exposure	78.4	[67.6,86.3]	21.6	[13.7,32.4]		83.5	[71.4,91.1]	16.5	[8.9,28.6]	
More than 1 type exposure	80.2	[78.3,82.1]	19.8	[17.9,21.7]		87.3	[86.3,88.4]	12.7	[11.6,13.7]	
Tobacco promotion exposure										
No exposure	87.4	[85.8,88.8]	12.6	[11.2,14.2]	0.000﻿	91.6	[90.7,92.5]	8.4	[7.5,9.3]	0.000﻿
One type exposure	75.2	[71.8,78.3]	24.8	[21.7,28.2]		80.7	[78.1,83.1]	19.3	[16.9,21.9]	
More than 1 type exposure	59.7	[55.3,63.9]	40.3	[36.1,44.7]		73.6	[69.5,77.2]	26.4	[22.8,30.5]	
Tobacco sponsorship exposure										
No exposure	87.2	[85.9,88.4]	12.8	[11.6,14.1]	0.000	90.1	[89.2,91.0]	9.9	[9.0,10.8]	0.000﻿
One type exposure	67.5	[63.7,71.1]	32.5	[28.9,36.3]		81	[77.1,84.3]	19	[15.7,22.9]	
More than 1 type exposure	59.7	[54.8,64.4]	40.3	[35.6,45.2]		77.3	[72.8,81.2]	22.7	[18.8,27.2]	

The accessibility to buy or consume cigarette variable showed that almost 20% of current smoker group and more than 10% of susceptible group were easy to buy cigarette. Further, the second hand smoking variables showed that in all places (in house and in school) significantly related to the outcome variables (*p* value: 0.001, < 0.01, < 0.05). In addition, the TAPS exposure variables showed similar result that all the type of TAPS exposure were significantly related to the outcomes variables (*p* value: 0.001, < 0.01, < 0.05).

Tobacco advertisements, as one of the components of TAPS, showed that the *p* value in each outcome variables were < 0.001 and the percentage of the exposure of more than 1 type of tobacco advertisements in each variable were 19.8% (current smoker) and 12.7% (smoking susceptibility).

The other components of TAPS were tobacco promotions, showed that the *p* value in both outcome variables were similiar (< 0.001) and the percentage of the exposure of more than 1 type of tobacco promotions in each variable were 40.3% (current smoker) and 26.4% (smoking susceptibility).

The last components of TAPS were tobacco sponsorships, showed that the *p* value in both outcome variables were similiar (< 0.001) and the percentage of the exposure of more than 1 type of tobacco sponsorships in each variable were 40.3% (current smoker) and 22.7% (smoking susceptibility).

The result also showed that the percentage of the exposure anti-smoking messages in all types to the outcome variables were not more than 30%. In the current smoker group, only anti-smoking messages at events exposed more than 25% of them. Meanwhile, the anti-smoking messages in media and school exposed less than 25% to the current smoker group.

In the smoking susceptibility group, the percentage of the exposure to anti-smoking messages were lower than 15% in all types. Anti-smoking messages on media and at events exposed around 13% to the smoking susceptibility group, while anti-smoking messages in school exposed around only 11% to the smoking susceptibility group.

Table 5 showed multivariate analysis of the relationship between the exposure of anti-smoking messages with current smoker variable and smoking susceptibility variable among Indonesian youth. The multivariate analysis conducted after controlling all covariates, which were characteristic of the respondents, accessibility to buy cigarettes, second hand smoking, and TAPS exposure.

**Table 5:** Multivariate analysis of the relationship between the exposure of anti-smoking messages with current smoker and smoking susceptibility

**Variables**	**Current Smoker (** ***n*** ** = 8,428)**				**Smoking Susceptibility (** ***n*** ** = 6,952)**			
	**OR**	**95% CI**	**AOR**	**95% CI**	**OR**	**95% CI**	**AOR**	**95% CI**
**Anti-Smoking Exposure**								
Anti-smoking messages on media (i.e.: television, radio, internet, billboards, posters, newspapers, magazines, or movies)								
No	Ref		Ref		Ref		Ref	
Yes	1.23	1.04—1.45	1.41**	1.15—1.73	1.12	0.90—1.39	1.24	0.93—1.64
Anti-smoking message at events (i.e.: sports events, fairs, concerts, or community events, or social gatherings)								
No	Ref		Ref		Ref		Ref	
Yes	1.84	1.57—2.17	1.26**	1.06—1.50	1.14	0.95—1.37	0.84	0.68—1.04
Anti-smoking messages in the school								
No	Ref		Ref		Ref		Ref	
Yes	0.98	0.82—1.16	0.96	0.78—1.17	0.93	0.77—1.13	0.92	0.73—1.13
**Grade**								
Junior High School	Ref		Ref		Ref		Ref	
Male	1.02	0.80—1.31	1.28	0.99—1.66	1.02	0.82—1.27	1.06	0.85—1.32
**Weekly spending money**								
More than Rp50,000	Ref		Ref		Ref		Ref	
Rp41,000-Rp50,000	0.98	0.75—1.27	0.76	0.56—1.05	1.00	0.76—1.31	0.89	0.67—1.19
Rp31,000-Rp40,000	0.87	0.64—1.18	0.67*	0.47—0.94	0.82	0.56—1.21	0.73	0.49—1.08
Rp21,000-Rp30,000	1.00	0.77—1.27	0.76	0.56—1.03	1.00	0.77—1.30	0.95	0.72—1.25
Rp11,000-Rp20,000	1.30	1.08—1.57	1.01	0.78—1.32	0.93	0.70—1.23	0.85	0.63—1.14
Less than Rp11,000	1.35	1.10—1.66	0.98	0.75—1.28	1.05	0.77—1.44	0.94	0.68—1.32
Usually don’t have any spending money	0.21	0.17—0.25	0.71	0.50—1.02	0.95	0.67—1.35	0.70	0.49—1.02
**Accessibility to buy or consume cigarette**								
Easy	Ref		Ref		Ref		Ref	
Difficult	0.91	0.77—1.08	0.87	0.72—1.07	1.21	1.05—1.39	1.22*	1.02—1.45
**Second hand smoking**								
Saw anyone smoking in the house								
Yes	Ref		Ref		Ref		Ref	
No	4.13	3.47—4.93	3.98***	3.23—4.91	1.46	1.21—1.77	1.32**	1.10—1.60
Saw anyone smoking in the school								
Yes	Ref		Ref		Ref		Ref	
No	1.68	1.45—1.95	1.30**	1.10—1.55	1.53	1.26—1.86	1.29*	1.06—1.57
Parents smoke tobacco								
Yes	Ref		Ref		Ref		Ref	
No	1.21	1.01—1.45	1.01	0.83—1.25	1.33	1.12—1.59	1.31**	1.09—1.56
**Tobacco Advertisement, Promotion and Sponsorship (TAPS) Exposure**								
Tobacco advertisement exposure								
No exposure	Ref		Ref		Ref		Ref	
One type exposure	2.41	1.40—4.17	1.94	0.94—4.03	2.67	1.26—5.62	2.21*	1.04—4.70
More than 1 type exposure	2.16	1.66—2.81	1.01	0.74—1.39	1.95	1.40—2.70	1.30	0.94—1.80
Tobacco promotion exposure								
No exposure	Ref		Ref		Ref		Ref	
One type exposure	2.29	1.92—2.73	1.33*	1.03—1.70	2.62	2.17—3.16	2.05***	1.69—2.48
More than 1 type exposure	4.69	3.91—5.62	2.41***	1.92—3.03	3.94	3.14—4.95	2.95***	2.35—3.70
Tobacco sponsorship exposure								
No exposure	Ref		Ref		Ref		Ref	
One type exposure	3.29	2.80—3.85	2.10***	1.74—2.54	2.14	1.69—2.70	1.68***	1.33—2.13
More than 1 type exposure	4.61	3.72—5.71	2.74***	2.01—3.74	2.68	2.08—3.45	1.96***	1.46—2.63

^***^ *p* < 0.001, ^**^ *p* < 0.01, ^*^ *p* < 0.05

Based on current smoker variables, adolescent who exposed to the two variables of anti-smoking messages increased the odds to become current smoker. The variables were anti smoking messages in media (AOR 1.41; 95% CI 1.15–1.73) and at events (AOR 1.26; 95% CI 1.06–1.50). Meanwhile, the exposure of anti-smoking messages in the school did not have relation with current smoker variables.

In the smoking susceptibility variables, there were no types of anti-smoking messages that were significantly related. It indicated that any type of anti-smoking messages had no relation with the susceptibility of smoking.

## Discussion

Our study showed that the result of our independent variables differed from majority of the researches. As a matter of fact, the exposure to anti-smoking media and at events increased the odds to become current smokers. While other independent variables were not significantly related to the outcomes variables. It meant that the anti-smoking messages needed to be modified so that the Indonesian youth understand and implement the important anti-smoking messages.

Our study found that anti-smoking messages in media and at event increased the odd to become current smoker and there were no relation with smoking susceptibility. It meant that the messages stimulated the youth to initiate or continue smoking cigarette. The findings were contrary to the majority researches that conclude any kind of media and program communication contributed to decrease the risk smoking behavior among youth group [21–23].

The result of anti-smoking messages in school variable showed slightly different from the other independent variables. Anti-smoking messages in school were not related to both outcomes variables. It meant that the messages were not effective enough to convey anti-smoking messages to the Indonesian youth. The findings were to some extent divers from the mostly previous study about anti-smoking campaign, particularly in the school. The previous study found that school were able to convey the anti-smoking messages, such as prevent and encourage youth not to initiate smoking cigarettes, the danger of smoking, etc [24, 25].

Based on the authors’s experiences in the tobacco control field, the situation above happened because of several causes, such as the tobacco industry interference (TII) and the intensity of TAPS exposure. TII is several actions designed by tobacco industry to tackle and interfere with tobacco control, some of which are direct and indirect political lobbying and campaign contributions, financing of research, attempting to affect the course of regulatory and policy machinery and engaging in social responsibility initiatives as part of public relations campaigns [26]. In 2021, the result of the Global Tobacco Index score in Indonesia was 83 and the rank was 77^th^ from 80 countries [27]. It means that TII in Indonesia was still high compare to other countries.

The intensity of TAPS exposure and youth smoking behavior in Indonesia was proven by several researchers. In 2016, Prabandari et al., conducted study about assessing the relation between youths’ perceive of cigarette advertising and smoking initiation. This study discovered that cigarette advertisements were considered as influencing teenagers to smoke, and that smoking status was consistently correlated with perception of youth-targeted cigarette advertisements, attitude toward TAPS, susceptibility, and smoking friends and family [28]. Sutrisno et al., in 2021 found that there was a strong association between cigarette advertisement exposure and smoking friends and adolescents’ intention to smoke in the immediate and long term in Sleman Regency, Indonesia [29]. In Malang, Indonesia, during the pandemic situation, Laili et al., found that youth were quite highly exposed to the various types of advertisements and promotions of cigarettes that can influence increased smoking behavior [30].

However, Indonesia had several campaign conveying the anti-smoking messages. National campaign that launched by Indonesian Ministry of Health in 2016 were *Suara Hati Anak* (the voice of children) [31, 32]. The campaign was taken from the true story of a smoker in Muara Angke, North Jakarta, who because of his smoking habit ended up ruining the lives of his children. The other national campaign was Suara Tanpa Rokok (voice without cigarette) [33, 34]. The campaign talked about health consequences from smoking cigarettes.

On the other hand, almost all of covariates statistically had relation with the outcome variables, particularly second hand smoking (saw anyone smoking in house, school, and parents smoking), and TAPS [35] exposure. These findings were similar with majority of the previous researches. Study conducted by Efendi et al., (2019) found that in rural Indonesia, a significant portion of young men and teenagers consume tobacco [36]. Vitoria et al., (2020) stated in their findings that if their parents smoked, the respondents (senior high school students) had a higher likelihood of smoking as well [37]. Nurmansyah et al., (2020) stated that the smoking behavior of the respondents (teenagers in City of Depok, Indonesia) were found to be highly influenced by peer (friend), family, and teachers who smoked [38].

This study showed that TAPS exposure variables were significantly related to the outcomes variables. The findings were in line with previous researches that TAPS in all kind and creative form were related to the youth smoking behavior. Several researchers found that tobacco advertising (such as TV, internet, billboard, etc.) were the determining factors to the smoking behaviour [39, 40]. Tobbaco promotion (such as free vouchers, free gift, etc.) also contributed to the smoking behavior in youth group, some researches found that the initiation to smoking cigarette due to the tobacco promotion [41, 42]. Tobacco sponsorship (such as in the sports events, community events, etc.) also were one of the factors that lead youth to initiate smoking cigarette [43, 44].

Researcheres and some agencies stated that to convey anti-smoking messages effectively we can use health communications as a strategy [45]. CDC formulated that there were four types of health communication strategy to convey anti-smoking messages, which were paid media, earned media, social media, and program communication. Anti-smoking messages contain many information about the prevention of smoking, smoking cessation, the danger of smoking, etc [46, 47]. Adult- and youth-targeted public education efforts can have a significant impact on youth by avoiding initiation to smoke and maintaining prevalence reductions [46].

### Study limitation

This study has a limitation: the study’s variable is limited and depends on the availability of secondary data (GYTS data). And also, the authors don’t have specific information in the some detail aspects, such as what is the main message in the anti-smoking campaign, what kind of internet the respondent use when see TAPS or anti-smoking messages. etc. However, the study has a positive impact on the tobacco control programs in Indonesia, particularly to prevent youth from smoking, and this study can be estimated to the national level with correct weight to describe the level of the problem at the national level. Furthermore this study can be used by other researches to investigate deeper aspect of anti-smoking campaign in Indonesia, such as, the messages, the program, etc.

## Conclusions

The study concluded that there were only two variables of the anti-smoking messages that had relation with the Indonesian youth smoking behavior, which were current smokers. Unfortunately those variables increased the odds of the respondents to become current smokers. Based on the findings of the research, we recommend to Indonesia government to develop a new anti-smoking messages following international best practices [21–23] in all type of channel of communication so that Indonesian youth can easily access to it and to counter tobacco advertisement, promotion and sponsorship.

## Data Availability

The datasets generated and/or analyzed during the current study are available in the CDC website repository, https://nccd.cdc.gov/GTSSDataSurveyResources/Ancillary/DataReports.aspx?CAID=2.
